# Refractory thrombotic thrombocytopenic purpura associated with oral contraceptives and factor V Leiden: a case report

**DOI:** 10.1186/1757-1626-2-6611

**Published:** 2009-05-12

**Authors:** Kostas Stylianou, George Tsirakis, Elpis Mantadakis, Irini Xylouri, Andreas Foudoulakis, Eleftheria Vardaki, Irene Katsipi, Eugene Daphnis, George Samonis

**Affiliations:** 1 Department of Nephrology and University Hospital of HeraklionHeraklion, CreteGreece; 2 Department of Haematology and University Hospital of HeraklionHeraklion, CreteGreece

## Abstract

**Introduction:**

Thrombotic microangiopathies constitute a heterogeneous group of diseases characterised by microangiopathic haemolytic anaemia and thrombocytopaenia associated with platelet aggregation in the microcirculation responsible for ischaemic manifestations. Classically, thrombotic microangiopathies are described as encompassing two main syndromes: thrombotic thrombocytopaenic purpura and the haemolytic-uraemic syndrome Many cases of idiopathic thrombotic thrombocytopaenic purpura have, to date, been associated with severe ADAMTS13 metalloprotease deficiency while haemolytic uraemic syndrome usually occurs in the context of normal protease activity. Oestrogens and factor V Leiden have rarely been implicated in the pathogenesis of thrombotic microangiopathy.

**Case presentation:**

We describe the case of a 17-year-old female with refractory thrombotic thrombocytopaenic purpura. The patient was receiving a new generation of oral contraceptives for dysmenorrhoea and had factor V Leiden. After undergoing prolonged and intense plasma exchange therapy for 40 days and high dose oral corticosteroids therapy for 90 days, our patient recovered fully.

**Conclusion:**

Patients with refractory thrombotic thrombocytopaenic purpura should likely be evaluated for congenital thrombophilic disorders and for ingestion of drugs that have been associated with this rare form of thrombotic microangiopathy. Identification of these and as yet other unknown genetic and/or acquired risk factors may lead to more judicious treatment approaches.

## Introduction

Thrombotic microangiopathy (TMA) is a syndrome caused by the development of hyaline thrombi in the microvasculature resulting in thrombocytopaenia, microangiopathic haemolysis, and organ dysfunction [1]. TMA includes: a) idiopathic thrombotic thrombocytopaenic purpura (TTP) with or without severe ADAMTS13 deficiency, the latter being either congenital or acquired due to an inhibitor; b) haemolytic uraemic syndrome (HUS), diarrhoea associated (epidemic or sporadic) or due to complement factors and regulatory protein alterations and c) secondary forms of TMA due to drugs, disseminated malignancy, pregnancy or postpartum, haematopoietic stem cell transplantation, autoimmune and other diseases with overlapping clinical manifestations [1,2]. As there is significant overlap these general categories are not accepted by all authors in the field and today the distinction between TTP and HUS probably describes more a phenotype than an underlying pathophysiology.

Autoimmune inhibitors or genetic mutations of the von Willebrand factor (VWF)-cleaving metalloprotease, ADAMTS13, result in unusually large VWF multimers that play a central role in the pathogenesis of TTP [1-4].

We present a case of a young woman with factor V Leiden (FVL), who was prescribed a new generation contraceptive and subsequently developed refractory TTP. Here we discuss the possible role of FVL and/or oral contraceptives in the development of TTP.

## Case presentation

A 17-year-old Greek Caucasian female was admitted to our department with weakness, new onset purpura, lethargy, and mild fluctuating dysarthria over the last 24 hours. She had recently followed a 10 days course of oral contraceptives (0.02 mg ethinylestradiol and 0.075 mg gestodene, Harmonette) because of dysmenorrhoea. One year earlier she reported having taken a similar compound for the same reason, with no negative side effects. There was no personal or family history of any gynaecological or haematological disease.

Vital signs on admission were normal. Physical examination revealed a purpuric rash on both legs, while the liver and spleen were not palpable. Laboratory findings on presentation were remarkable for decreased haemoglobin, 101 g/L, marked thrombocytopaenia, 15×10^9^/L, elevated lactate dehydrogenase (LDH), 599 IU/L, and elevated indirect bilirubin, 43 μmol/L. Coagulation tests were normal. Haemolytic anaemia was confirmed by low serum haptoglobin, <0.1 g/L and schistocytes on blood smear compatible with TTP. Direct Coomb’s test was negative, and clotting times were normal. Serum creatinine and liver function tests were within normal limits. Lupus anticoagulant, anti-dsDNA antibodies, as well as anti-phospholipid antibodies were negative. Urinalysis revealed haematuria of glomerular origin and trace of protein.

She was immediately commenced on daily, single volume (2.5 Lt) PE with fresh frozen plasma and 100 mg per day oral prednisone. The platelet count and the LDH levels normalized during the first 5 days of therapy. However, on the 7^th^ day of daily plasmapheresis, the platelet count decreased to 20×10^9^/L, while LDH increased to 802 IU/L. At that point twice-daily single volume PE was empirically instituted with continuation of the corticosteroid therapy. She responded slowly and recovered normal PLT levels 10 days after the beginning of intense plasmapheresis. On the 17^th^ day, the patient was weaned off plasmapheresis to daily single volume PE, which resulted in a second drop of PLTs to 100×10^9^/L necessitating intensification of steroid and PE treatment for a second time. She finally received 42 sessions of PE within 40 days and was weaned off corticosteroids after 90 days of treatment.

Due to the prolonged course of her illness a screen for genetic polymorphisms associated with thrombophilia such as prothrombin G20210A, methylenetetrahydrofolate reductase (MTHFR) C677T and factor V G1691A (FVL) was requested, which only disclosed FVL heterozygous status. On discharge she was advised to avoid oral contraceptives. Four years later, TTP has not relapsed and the patient remains in excellent clinical condition.

## Discussion

Many drugs have been implicated as aetiological factors in the pathophysiology of TMA. Oestrogens have been associated with acquired idiopathic TTP, probably by reducing prostacyclin production [5,6]. The particular contraceptive that our patient received has not been previously reported to cause TTP. Nevertheless it is known that all natural and synthetic forms of oestrogens can induce TTP, including contraceptives [5,7,8], tamoxifen [9], hormonal replacement preparations [10,11] or increased production of oestrogens due to pregnancy or post-partum [7,12]. Whether the concurrent use of progesterone protects [5] or triggers the disease [13] is controversial. Risk factors include positive family history, concurrent toxin exposure and recently FVL [14]. Most cases of oestrogen-related TTP seem to be idiosyncratic, although re-exposure may cause relapses [5]. Our case illustrates some of the characteristics of oestrogen related TTP. Firstly, it occurred in a patient with previously uncomplicated exposure, probably as a result of sensitization of the immune system. Secondly, it was difficult to manage, with refractoriness to plasmapheresis. Refractory TTP is defined in the presence of persistent thrombocytopaenia (platelets < 150×10^9^/L) or LDH elevation after a total of seven daily plasma exchanges [15]. Thirdly petechiae presented few days after the patient had received contraceptives. However, the rarity of TTP in recipients of oral oestrogens shows that exposure to oestrogens alone is not sufficient for the development of the syndrome. Other predisposing factors seem to be necessary which in our case may have been FVL.

Inactivation of factor Va by activated protein C is impaired in persons with FVL, and the increased prevalence of this thrombophilic disorder in some patients with TMA is consistent with studies that have implicated the thrombomodulin-protein C anticoagulant pathway in TMA. In a study by Raife et al [14] the prevalence of FVL was significantly increased among Caucasian TMA patients with normal ADAMTS13 activity. Different results have been reported by Krieg et al [4] who determined ADAMTS13 activity and FVL carriership in 256 patients presenting with various forms of TMA [4]. FVL was equally common in patients with (6.8%) or without (8.6%) decreased ADAMTS13 activity. However a higher prevalence of FVL (12.3%) was noticed in patients with HUS and normal ADAMTS13 activity and therefore a contributory role of FVL could not be excluded in that study. A recent registry study from South East England showed that ADAMTS 13 activity levels were not significantly different between idiopathic and secondary forms of TTP [3]. It is also known that although ADAMTS13 levels are prognostic of future relapses, the response rates to PE are similar for idiopathic TTP with or without severe ADAMTS13 deficiency [1,2]. Therefore modifying factors may impact on the severity of the disease and the response to treatment. In a study by Wada et al [16] the plasma protein C and antithrombin activities were markedly reduced in TTP patients who died compared to those who survived, suggesting that reduced plasma antithrombin and protein C activities can be prognostic of outcome. Therefore resistance to activated protein C due to FVL may have a similar impact on outcomes. Most studies examining FVL prevalence in TTP were restricted to exploring a possible pathogenetic role of FVL in patients with normal ADAMTS13 activity, whereas they did not examine a possible role on the severity of the clinical manifestations and the response to treatment. We do not propose performing FVL testing on every patient presenting with acute TTP, but certainly in refractory cases such information may help in avoiding early PE withdrawal and consequent early relapses.

We did not measure the serum VWF-cleaving protease activity in our patient, since the necessary assays were not available. The diagnostic possibilities in our patient were either idiopathic TTP or secondary TTP due to oral contraceptives. In either case the possibility of having ADAMTS13 deficiency would be high and therefore, the differential diagnosis could not have been based on ADAMTS13 levels. Moreover immediate clinical management would not have been modified by the results of these assays.

In conclusion, we present a case of refractory TTP in a young woman who had been receiving a new generation oral contraceptive and had concurrent FVL. We suggest that patients with refractory TTP should likely be investigated for ingestion of incriminating drugs and for congenital thrombophilic disorders.

Identification of these and other possible applicable constitutional or acquired risk factors could lead to more individualized treatment approaches for patients with TMA.

## List of abbreviations

FVL: Factor V Leiden
HUS: Haemolytic-uraemic syndrome
LDH: Lactate dehydrogenase
MTHFR: Methylenetetrahydrofolate reductase
PE: Plasma exchange
PLT: Platelet Count
TMA: Thrombotic microangiopathy
TTP: Thrombotic thrombocytopaenic purpura
VWF: Von Willebrand Factor
